# Eosinophils in Rheumatoid Arthritis: A Multifaceted Role in the Pathogenesis of the Disease

**DOI:** 10.32604/biocell.2025.062821

**Published:** 2025-07-25

**Authors:** Alexander Blagov, Michael Bukrinsky, Aleksandra Utkina, Gulalek Babayeva, Vasily Sukhorukov, Alexander Orekhov

**Affiliations:** 1 Laboratory of Angiopathology, Institute of General Pathology and Pathophysiology, Moscow, 125315, Russia; 2 School of Medicine and Health Sciences, The George Washington University, Washington, DC 20037, USA; 3 Department of Commodity Expertise and Customs Business, Plekhanov Russian University of Economics, 36, Stremyanny Lane, Moscow, 115054, Russia; 4 Institute of Experimental Cardiology, National Medical Research Center of Cardiology, 15A 3-rd Cherepkovskaya Street, Moscow, 121552, Russia; 5 Faculty of Biology and Biotechnology, National Research University Higher School of Economics, 33, Profsoyuznaya Street, Building 4, Moscow, 117418, Russia

**Keywords:** Rheumatoid arthritis, inflammation, eosinophils, cytokines, lymphocytes, synovium

## Abstract

Eosinophils are multifunctional granulocytes that contribute to the initiation and modulation of inflammation. Accumulating evidence suggests that eosinophils are adaptable leukocytes that orchestrate the resolution of inflammatory responses. The most prevalent chronic inflammatory illness, rheumatoid arthritis (RA), is typified by persistent synovitis that makes it hard for the disease to go away on its own. Interestingly, a unique subset of eosinophils known as regulatory eosinophils has been found in RA patients’ synovium, especially while the disease is in remission. Pro-resolving signatures of regulatory eosinophils in the synovium are distinct from those of their lung counterparts. The most recent research on eosinophils and their function in this disease pathogenesis is compiled in this review. Based on the role of regulatory eosinophils, a new pathological model of inflammation resolution in RA is proposed, and potential therapeutic strategies aimed at enhancing the action of regulatory eosinophils in RA are proposed.

## 1 Introduction

Inflammatory arthritis and extra-articular intervention are hallmarks of rheumatoid arthritis (RA), which is a systemic autoimmune illness. It is a chronic inflammatory illness that mostly affects the synovial joints and is frequently brought on by the interaction between genetic and environmental factors, particularly tobacco [1,2]. If treatment is not received, disease usually starts in small peripheral joints and eventually spreads to proximal joints [3]. Over time, joint inflammation causes bone erosion and cartilage loss, which results in joint deterioration. Early RA is defined as having symptoms for less than six months, whereas established RA refers to symptoms persisting for more than six months. If left untreated, RA becomes a progressive illness with high morbidity and mortality [4].

There is a substantial genetic component to the genesis of RA. It is believed to be caused by the interaction between environmental influences and patient genotypes. The most potent environmental risk factor for rheumatoid arthritis is cigarette smoking. Research has demonstrated that smoking and the shared epitope (SE) combined in patients’ anamnesis raise the risk of RA in those who screened positive for anti-citrullinated protein (anti-CCP) antibodies [5].

Estimated 0.24% to 2% of people globally have RA [6]. In North America, Western Europe, Scandinavia, and other areas with a European ancestry, such as Australia, the frequency of RA is greater [7]. In South and Central America, as well as in East Asia and Africa, the frequency is significantly lower [7]. Epidemiological data indicate that women are more likely than men to get RA, with a lifetime prevalence of 3.6% for women and 1.7% for men. RA incidence peaks between the ages of 65 and 80, with vulnerability increasing with age [7].

Early diagnosis of rheumatoid arthritis is challenging since there is no pathognomonic testing method for this condition [8]. To make a diagnosis and avoid crippling joint injury, a thorough clinical approach is necessary. Both pharmaceutical and non-pharmacologic therapies are necessary for the treatment of rheumatoid arthritis patients [9]. Early therapy with disease-modifying antirheumatic medications is now the accepted standard of care [10]. Nevertheless, many individuals have severe morbidity and eventually become disabled despite therapy [1].

Innate immune system cells may contribute to autoimmune diseases. Potential roles of innate immune cells exist not only in the initial stage of autoimmune diseases but also in the modulation and propagation of inflammation and tissue destruction [11]. Such roles have been proposed for neutrophils, natural killer cells, macrophages, dendritic cells, innate lymphoid cells, and mast cells [12]. Eosinophils have been recognized as part of the inflammatory infiltrate in patients’ serum samples who have different autoimmune diseases, including RA [13]. However, their potential role has not been fully explored. This review aims to define criteria for the influence of eosinophils on the development of RA and to propose therapeutic approaches for the treatment of RA that target eosinophils.

## 2 Structure and Functions of Eosinophils

Eosinophils typically have a diameter of 10–16 μm with a segmented, lobulated nucleus [14]. Numerous molecules with pleiotropic activities, including lipid mediators, growth factors, chemokines, cytokines, cationic granule proteins, and immunomodulatory compounds, are present in these cells and primarily accumulate in the intracellular compartment [15]. In addition, a wide variety of surface receptors and transmembrane proteins (integrins) are present in eosinophils, which facilitate interaction with the cellular environment and enable response to various stimuli [16].

Different kinds of granules are present in the cytoplasm of eosinophils. Specific granules and premature specific granules are the two primary forms of big granules found in mature human eosinophils. Specific granules, also known as “secondary granules”, are composed of a membrane enclosing a tight crystalline core [17]. These granules carry a wide range of mediators that can induce tissue damage and inflammation. These mediators include growth factors, cytokines, chemokines, and basic proteins. The primary particular granule compounds that are displayed are neurotoxic, which are eosinophil peroxidase (EPX), eosinophil-derived neurotoxin (EDN), major basic protein (MBP), and eosinophil cationic protein (ECP) [18]. Generally smaller than specific granules, specific immature granules are also referred to as “primary granules” and are the major site of the Charcot-Leyden crystal protein (a representative of the galectin-10 family that binds carbohydrates). Lipid bodies, a third intracellular compartment identified exclusively in eosinophils, are known to be produced to generate eicosanoid inflammatory mediators [19].

Eosinophils have a wide range of pleiotropic activities, including protective immunity and antimicrobial activity, and are responsible for several physiological responses such as organ development and metabolism [20]. Although eosinophils are generally considered circulating blood cells, a proportion of eosinophils are stably resident in various tissues [21]. Mainly presented in a number of mucosal regions, tissue-resident eosinophils support a range of homeostatic and tissue-protective processes [22]. In the control and modification of immune responses, eosinophils have important functions. Eosinophils have a significant non-inflammatory role in the development and sustenance of adaptive immunity. Both the lengthy survival of plasma cells in the bone marrow and the sustenance of these cells in the lamina propria underneath the intestinal epithelium depend on eosinophils, which are the primary source of the plasma cell survival factor APRIL (activation and proliferation-inducible ligand) [23]. Additionally, there is evidence that eosinophils are required for tissue regeneration and for maintaining tissue integrity. It has also been shown that these cells played a part in the production of B cells that produce IgA and, consequently, in the creation of IgA plasma cells that live in the lamina propria and support immunological protection at mucosal membranes [24].

Numerous allergies, rheumatological, infectious, neoplastic, and uncommon idiopathic diseases are linked to eosinophil buildup in blood and tissues, which can also control local immunological and inflammatory responses [25]. The involvement of eosinophils in the pathophysiology of eosinophil-associated disorders has been the subject of several investigations, despite the fact that they can support tissue homeostasis under a steady state [26]. In fact, eosinophils can use different mediators such as chemokines, lipid mediators, proinflammatory cytokines: tumor necrosis factor alpha, interleukins-1b, 6 and 8 (TNF-α, IL-1b, IL-6, and IL-8), type 1 cytokines: interleukin-12, interferon gamma (IL-12, IFN-γ), and type 2 cytokines: interleukins-4, 5, 9, 13 and 25 (IL-4, IL-5, IL-9, IL-13, and IL-25) to carry out their biological activities [27]. Eosinophils have been found to be significantly associated with certain disorders that are defined by inflammation. Tissue inflammation is caused and maintained by the activation of eosinophils and the production of proinflammatory lipoprotein mediators, cytokines of the acute phase, different forms of free oxygen radicals, and highly charged cationic polypeptides. Furthermore, a malfunction in the apoptotic death of eosinophils has been linked to their accumulation in the bloodstream and tissues [28].

## 3 General Pathogenesis of RA

The precise origin of RA remains unknown, even though it is one of the most prevalent inflammatory arthritis types and has long been extensively researched as a prototype of autoimmune disorders. The occurrence and susceptibility to RA are influenced by both environmental and genetic variables. The pathophysiology of RA includes immunological aberrations, inflammatory pathways, genetic and epigenetic alterations, and metabolic disruptions [29].

### 3.1 Immunological Basis of RA

The pathophysiology of this condition involves a wide range of biological responses, such as local growth factors, inflammatory cell activation, cytokine production, and angiogenesis. The extracellular matrix (ECM) of cartilage and bone is destroyed by the inflammatory and degradative chemicals produced by T cells, B cells, neutrophils, and macrophages, which are primarily found in synovial tissue [30]. Patients with RA have chronic inflammation as a result of a cell-mediated immune reaction [31]. There are a lot of CD4+ T cells in the rheumatoid synovium, and it is believed that an antigen triggers CD4+ T cell activation, which suggests that T cells have a pathogenic role in this joint disease [32]. T lymphocytes, macrophages, and fibroblasts are considered to interact to cause the ongoing inflammation associated with RA [33]. An important stage in the onset of autoimmune diseases is the loss of self-tolerance and the initiation of naive antigen-specific T cells by antigen-presenting cells, especially dendritic cells [34].

B cells may be involved in the pathophysiology of rheumatoid arthritis in several ways, including the generation of autoantibodies, the association of other inflammatory cells, the initiation of pro- and anti-inflammatory cytokines, and T cell activation through the expression of costimulatory molecules. Joint destruction results from severe synovitis in inflamed joints, as well as from the erosion of nearby bone and cartilage [35]. Neutrophils secrete a large amount of proinflammatory cytokines, which is one of the reasons that affects tissue damage [36]. Matrix metalloproteinases (MMPs), a particular class of protein-degrading enzymes that may break down ECM proteins, are responsible for breaking down connective tissue [37]. MMPs are typically stimulated to produce more of these enzymes. Matrix metallopeptidase 3 (MMP-3) is among the most prevalent MMPs in RA patients’ synovial fluid and membrane [38].

### 3.2 Proinflammatory Cytokines

Patients experience the development of clinical symptoms as a result of the expansion and stimulation of synovial and epidermal fibroblasts induced by the secretion of cytokines by activated T cells and B cells [34]. Serum cytokines are generally produced in a cascade, whereby a particular cytokine stimulates target cells to produce further cytokines, and are well-known to play a crucial role in pathogenesis by starting and maintaining both humoral and cellular autoimmune branches of the immune system [39]. TNF-α and IL-1β, two proinflammatory cytokines, activate chondrocytes, osteoclasts, macrophages, and synovial fibroblasts [40]. MMPs that degrade ECM, specifically MMP-1 and MMP-3, are produced by these synovial cells and are implicated in tissue breakdown processes such as cartilage destruction [30].

Granulocyte-macrophage colony-stimulating factor (GM-CSF) and IL-1 are thought to be autocrinally stimulated and paracrinally induced by TNF-α [41]. As a result, TNF-α increases from its synthesis by positive responses to gene expression [42]. The promotion of joint inflammation associated with RA is known to be facilitated by TNF-α, as demonstrated by the reduction of inflammation caused by TNF-α neutralization [43]. Among the strongest proinflammatory cytokines, IL-1 is essential for joint bone and cartilage degradation and inflammation. It causes the increasing expression of adhesion molecules like vascular cell adhesion protein 1 (VCAM-1) and intercellular adhesion molecule 1 (ICAM-1) by promoting the production of IL-6, IL-8, and GM-CSF [44]. One of the key players in the pathophysiology of this illness is the pleiotropic cytokine interleukin-10 (IL-10), which promotes B cell viability, proliferation, differentiation, and antibody isotype switching [45]. Elevated amounts of TNF-α, IL-1β, and IL-10 were discovered in the blood of the individuals with RA [45]. Because the gene products of these cytokines are implicated in the pathophysiology of this illness, cytokine gene polymorphisms may be important genetic predictors of clinical outcome or disease susceptibility [46]. The variation in cytokine production levels determines the severity of RA [47,48].

### 3.3 Oxidative Stress

Antioxidants and oxidative stress are important factors in the pathophysiology of RA. Oxidative stress upsets the delicate balance between the cellular antioxidant system and the production of reactive oxygen species (ROS), damaging essential cellular constituents including proteins, DNA, and membrane lipids [49]. The production of ROS causes lipids and DNA to oxidize, which produces a variety of harmful chemicals, including lipid hydroperoxides, DNA, and alkanals [50]. Lipid peroxidation is shown to be much greater, and non-enzymatic antioxidant vitamin C is reduced significantly in individuals with RA [51].

### 3.4 Metabolic Disorders in RA

Researchers have recently focused a lot of emphasis on the energy metabolism pathways in rheumatic illnesses. Glycolysis, tricarboxylic acid cycle, pentose phosphate pathway (PPP), fatty acid oxidation, fatty acid synthesis, and amino acid metabolism are the six main metabolic processes that are implicated in many aspects of the course of RA, such as the activation, proliferation, and differentiation of synovial cells [52].

According to metabolomics research, RA patients had lower levels of high-density lipoprotein cholesterol and higher levels of serum cholesterol [53]. As a result, scientists have looked at lipid metabolism as a way to regulate cardiovascular issues in RA. Clinical testing also often reveals alterations in fatty acids (FA) in RA patients [54]. By altering membrane permeability and lipid raft creation, lipid reprogramming influences proinflammatory signaling pathways [54]. Eicosapentaenoic acid (EPA) and docosahexaenoic acid in n-3 polyunsaturated fatty acids (PUFA) are thought to be mediators that cause inflammation resolution, whereas arachidonic acid in n-6 PUFA and its derivatives are primarily thought to be proinflammatory mediators [55]. Furthermore, lipoxins, E-series resolvins, D-series resolvins, protectins, and maresins are examples of the specific pro-resolving mediators (SPMs) that are crucial for the regression of inflammation [56]. Both higher levels of arachidonic acid-derived leukotriene B4 (LTB4) and lower levels of SPMs have been observed in the serum and synovial fluid of RA patients [57].

The plasma of RA patients also has changed amounts of total amino acids, with glutamic acid, kynurenine (Kyn), and homoserine dramatically increasing and alanine, histidine, arginine (Arg), valine, serine, tryptophan (Trp), lysine, glycine, arginine, and creatinine greatly decreasing [58]. RA has been linked to indoleamine 2,3-dioxygenase 1 (IDO1), a crucial rate-limiting enzyme in the kynurenine pathway. IDO1 converts Trp to Kyn, which mitigates Immunity by initiating the aryl hydrocarbon receptor (AhR) via Kyn and decreases it by depleting Trp [59]. Arginase and nitric oxide synthase (NOS) both use Arg as a substrate. While ARG1 activity in M2 macrophages primarily causes Arg deprivation and hence has immunoregulatory effects on tissue repair, NOS2 in M1 macrophages transforms Arg into nitric oxide (NO) and L-citrulline during inflammation [60]. Research indicates that RA patients’ immune cells overexpress arginases in an effort to control inflammation and that arginases’ enhanced absorption of Arg may dramatically lower NO levels and increase the incidence of cardiovascular manifestations linked to RA [61].

## 4 Participation of Eosinophils in the Pathogenesis of RA

An evolutionary host defensive response to the damage, inflammation, is defined by the migration of circulating leukocytes and cytokines to the site of inflammation. Generally, acute inflammation in healthy persons is self-limited and resolves immediately, hence limiting the development to chronic inflammation [62]. Numerous autoimmune and inflammatory disorders in humans, including RA, are believed to have their origins in unchecked or protracted inflammation. According to recent investigations, eosinophils use the 12/15-LOX-mediated biosynthetic route to produce pro-resolving lipid mediators, which aid in the resolution of inflammation [63].

### 4.1 Regulatory Eosinophils in RA

Even though eosinophils function as counter-regulators in several inflammatory disorders, there is no evidence linking eosinophils to the onset of RA. This is probably because eosinophilia in RA presents peculiarly clinically [64]. The idea that eosinophils are engaged in the inflammatory responses of RA is supported by indirect evidence from earlier research showed higher serum ECP levels in RA patients, particularly those with high disease severity and brief illness duration [64].

It was recently demonstrated that individuals with RA had higher levels of synovial EP*X* expression than patients with osteoarthritis (OA) [65]. Accordingly, blood EP*X* levels were greater in RA patients than in healthy subjects. IL-5 transgenic (IL-5tg) mice, with remarkable hypereosinophilia, demonstrated a substantial decrease in arthritis scores in a serum-induced arthritis model of K/BxN, while eosinophil-deficient animals had increased disease activity [65]. Additionally, the adoptive introduction of eosinophils into mice that had arthritis caused by collagen led to an improvement in the arthritis, along with a reduction in bone erosion and joint inflammation as determined by histology [66]. These findings imply that eosinophils possess pro-resolving abilities that were previously unidentified and that aid in the healing of inflammatory arthritis.

Since eosinophils are both pro-inflammatory and pro-resolving cells, it seems sensible to think that distinct subsets of eosinophils are involved in various biological processes. In fact, research that examined two different eosinophil subsets in asthmatic lungs—lung resident eosinophils and recruited inflammatory eosinophils—supported this [67]. Notably, a relatively recent study identified a unique eosinophil population known as regulatory eosinophils (REs) that had been found in the synovium of RA patients as well as in the joints of arthritic mice [68]. Subsequent investigations using proteome profiling and single-cell RNA sequencing verified that the pro-resolving signature of REs in the joint had been different from that of their lung equivalent. Articular REs, for example, have significantly increased expression of 12/15-LOX [68], which may contribute to the anti-inflammatory response function of REs, as deletion of 12/15-LOX has been associated with unmanaged inflammatory response and tissue degeneration in chronic arthritis [69]. Further evidence that REs may not only prevent inflammation but also facilitate synovial tissue repair comes from the fact that they secrete differently in the lung than their “inflammatory cousins”, as evidenced by the synthesis of MMP-3, osteopontin, and serpin E1 [68]. Interestingly, compared to individuals in an active stage, RA patients in remission had higher rates of REs infiltration. Following anti-IL-5 monoclonal antibody therapy, patients with inactive RA and concomitant asthma had an exacerbation of disease, which is logically clarified by REs loss [68].

### 4.2 ILC2–Eosinophil–Macrophage M2 Pathway

It has been established that innate and adaptive immune responses in lymphoid cells (ILCs) play a key role in the pathophysiology of inflammatory arthritis by acting as a mediator between the innate and adaptive immune responses [70]. Specifically, ILC2 is a key regulator of the inhibition of joint inflammation, whereas pro-inflammatory ILC1/ILC3 is the opposite. In individuals with RA, the quantity of circulating ILC2s rose following antirheumatic medication and was inversely linked with the disease activity index [71]. Adoptive transfer of ILC2s from wild-type mice alleviated arthritis, but genetic deletion of ILC2s in mice exacerbated arthritis, by these human results [72]. Moreover, it was discovered that ILC2s, independent of inflammation, had suppressed osteoclast development and bone loss [71].

It should be attention that constitutive IL-5 production by tissue-resident ILC2 has been demonstrated to control eosinophil homeostasis and tissue accumulation. ILC2 was the primary IL-5 producer in asthmatic lungs, and IL-5, in turn, increased EC growth and invasion into arthritic joints [73]. A recent study showed that the advancement of collagen-induced arthritis, which had been associated with eosinophil growth in arthritic joints, had been considerably inhibited upon ILC2 activation by a neuropeptide [73]. Moreover, the injection of IL-25/IL-33 induced ILC2 and sped up the resolution of arthritis [73]. On the other hand, EC growth in joints was decreased when a monoclonal antibody neutralized IL-5, preventing the resolution of asthma-induced arthritis. When combined, these findings provide credence to the hypothesis that eosinophils have a significant involvement in the resolution of ILC2-mediated arthritis [73].

In addition to releasing a variety of pro-resolving lipid mediators that are critical for the resolution of inflammation, eosinophils may also be involved in the suppression of arthritis by causing macrophages to change from the pro-inflammatory M1 phenotype to the anti-inflammatory M2 phenotype [74]. It is commonly recognized that synovial macrophages play a key role as effector cells in the progression of synovitis [74]. The inflammatory synovium’s high concentration of pro-inflammatory cytokines, including TNFα, IL-6, and IL-1β, points to the M1 macrophage phenotype being prominent in RA.

On the other hand, synovial macrophage phenotype *in vivo* is very variable and frequently exhibits a combined polarization phase [75]. According to earlier reports, M1 macrophages induced during the early stages of arthritis can change their phenotype and become M2 macrophages [64]. M2 macrophages suppress joint inflammation by eliminating dead cells (efferocytosis), generating pro-resolving lipid mediators, and creating anti-inflammatory cytokines, including IL-10, IL-13, and TGF-β, in contrast to M1 macrophages that stimulate the inflammatory signal cascade in the synovium [65]. Eosinophils have been demonstrated to secrete lipid mediators generated from IL-4, IL-13, and 12/15-LOX, which polarized macrophages toward the M2 phenotype [76]. Impaired anti-inflammatory macrophage dispersion has been linked to eosinophil deficit [76].

Studies conducted *in vitro* and *in vivo* have demonstrated that eosinophils, partially via the I*κ*B/P38 MAPK signaling pathway, increased M1 to M2 macrophage polarization in synovial tissue [66]. This is in line with other research showing that the M2 phenotype of macrophages, which is necessary for glucose homeostasis, is mediated by adipose tissue eosinophils [77]. When combined, the ILC2-eosinophil-macrophage M2 pathway constitutes a unique and significant immune mechanism that reduces inflammation in the joints and promotes the resolution of arthritis. At the same time, it is necessary to highlight that the development of general eosinophilia in RA does not depend on the pathogenesis of RA, which manifests itself as a concomitant clinical symptom associated with helminthic infection or allergies [78].

**Figure 1:**
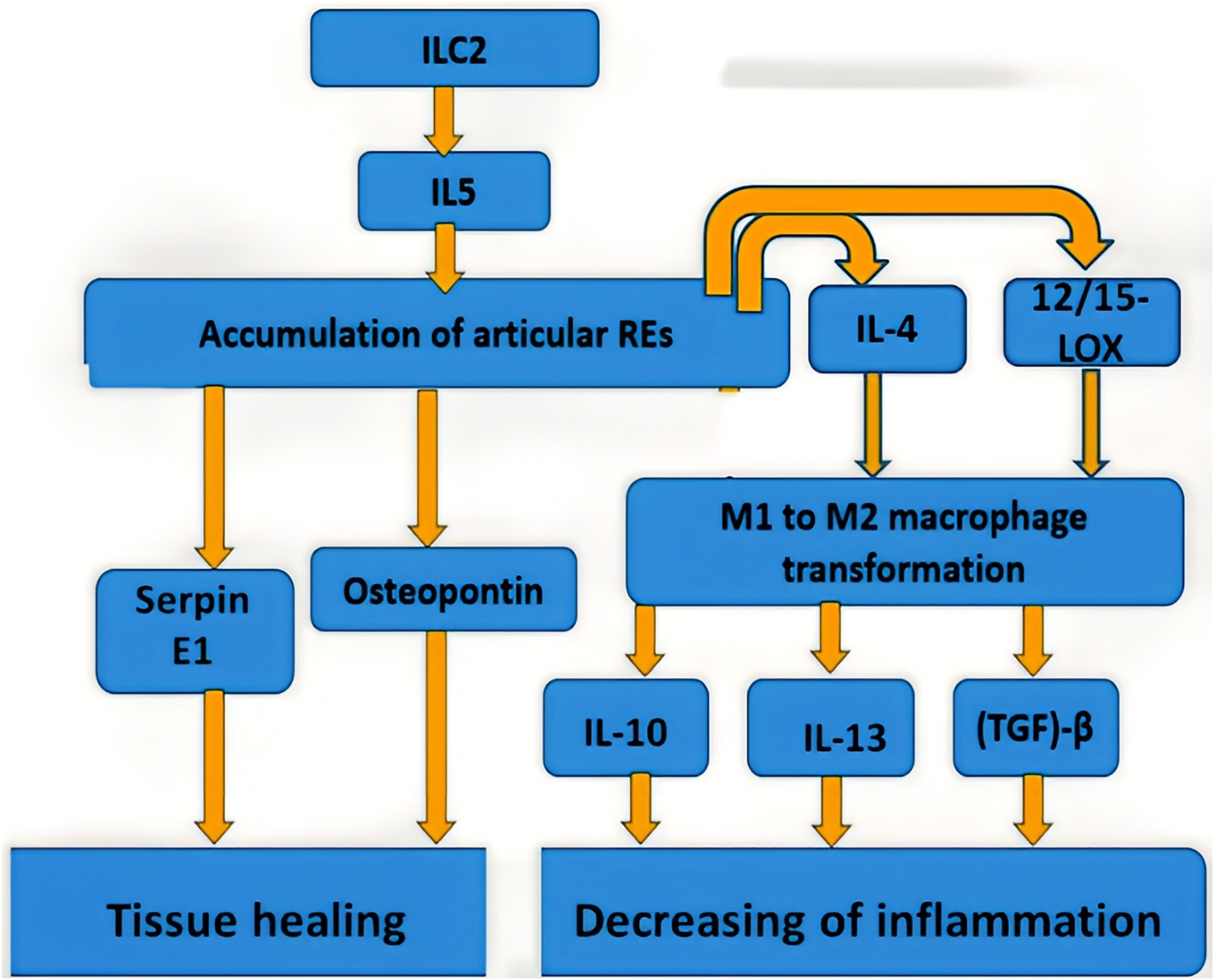
The general model of eosinophil participation in RA

The general model of eosinophil participation in RA is shown in Fig. 1.

## 5 Therapeutic Targets Aimed at Regulatory Eosinophils in RA

Based on the above model of eosinophil involvement in RA pathogenesis, it has been proposed that potential therapeutic targets, by influencing which the effect of REs can be enhanced, which can contribute to the improvement of RA symptoms. Here, four therapeutic strategies are proposed: the first is aimed at dominant eosinophil receptors, the second is aimed at anti-inflammatory cytokines that modulate the action of eosinophils, the third is aimed at the main mediator associated with the resolution of inflammation (RA—12/15-LOX), the fourth is aimed at mediators released by REs and associated with joint healing in RA. It should be noted that these strategies are suggestions by the authors of this review for researchers and clinicians working in this area. None of these strategies has been tested as intended in appropriate preclinical and clinical trials.

### 5.1 Eosinophil Cell Receptors (CCR3)

Highly expressed receptors on the cell surface of eosinophils that initiate their activation may potentially represent promising therapeutic targets. These receptors must be expressed predominantly on eosinophils, since secondary activation of other immune cells may lead to the development of serious adverse effects (AEs) or even contribute to the progression of RA. CC chemokine receptor 3 (CCR3) is one of these receptors. It should be taken into account that CCR3 is expressed on all types of eosinophils, not just REs, so therapeutic agents that enhance its function are not suitable for patients with concomitant allergic diseases, including bronchial asthma.

One of the treatment options may be the use of C-C motif chemokine ligand 11 (CCL11) mimetics, which activate CCR3. In a study [79], during the screening of small molecules, CCL11 mimetics were identified: CH0076989 and UCB35625, which had a Tyr-113 to Ala mutation, both of them bound to CCR3 with high specificity and caused a chemotactic response in eosinophils.

### 5.2 Anti-Inflammatory Cytokines (IL-5)

Anti-inflammatory cytokines, as already shown in the previous section, play an important role in the activation of eosinophils and subsequent cascades of reactions leading to the resolution of inflammation in the joints. In this case, one of the central roles is played by IL-5, which promotes the maturation and activation of eosinophils (see Fig. 1). The use of therapeutic compounds that enhance the action or expression of IL-5 will potentially contribute to better activity of REs in RA. However, in this case, it should also be remembered that this therapy is not suitable for patients with allergic reactions. It is known that IL-5 expression is enhanced by the synergistic action of acetyltransferase CREB-binding protein (CBP/p300) and transcription factors: CCAAT-enhancer binding proteins (C/EBP), nuclear factor of activated T-cells (NF-AT), and activator protein 1 (AP-1) [80]. In a mouse model, the effective use of the small molecule compound TTK21 in activating CBP/p300 was shown [81]. This model was used to study the treatment of neurodegenerative diseases, and the use of TTK21 in a suitable animal model of RA may provide insight into the potential of this strategy for the treatment of RA.

### 5.3 12/15-LOX

Since, as described in the previous section, 12/15-LOX has increased expression in REs and its loss correlates with the development of inflammation in the joints, this mediator is the most promising therapeutic target. High expression in REs, unlike other eosinophils, contributes to a low risk of developing severe AEs, primarily those associated with allergies. Direct participation of 12/15-LOX in the resolution of inflammation in RA is a prerequisite for the creation of highly effective therapies aimed at enhancing the function of 12/15-LOX.

An example of an effective activator of 12/15-LOX is 3-O-acetyl-11-keto-β-boswellic acid (AKBA), which activated 12/15-LOX through an allosteric site, which promoted the release of SPMs by immune cells [82]. Another approach may involve regulating the expression of 12/15-LOX. A study [83] demonstrated that inhibition of Lysine-specific demethylase 5C (KDM5C), which was a transcriptional repressor of 12/15-LOX, using shRNA resulted in increased expression of 12/15-LOX.

### 5.4 Healing Mediators: Osteopontin and Serpin E1

Secretion of healing mediators such as osteopontin and serpin E1 is one of the final steps in the inflammation resolution cascade in RA. Enhancement of their activity can potentially promote tissue healing in the area of inflamed joints in RA. The review [84] described several studies in which an exogenous osteopontin administration had resulted in improved neurological function and decreased inflammation in a rat model of traumatic brain injury. In the study [85], it was unexpectedly found that the purinergic inhibitor P2Y12 had been promoting increased serpin E1expression, which would create further prerequisites for analyzing its efficacy in an animal model of RA. Since high levels of serpin E1 are associated with thrombophilia, therapy aimed at enhancing its expression will not be suitable for patients with this disease.

Potential therapeutic strategies targeting eosinophil activation in RA are summarized in Table 1.

## 6 Discussion

The traditional understanding of eosinophils has evolved due to mounting data and technological advancements, moving away from pro-inflammatory cells in helminthiasis and allergies and toward a cell type that is actively engaged in anti-inflammatory reactions in the resolving of systemic inflammation. The knowledge that eosinophils are essential for the reduction of joint inflammation and acceleration of the resolution of the illness has been broadened by the discovery that regulatory eosinophils are present in the synovium of RA patients [68]. Given that existing medications target pro-inflammatory cytokines and mediators instead of supporting inflammation resolution, these findings are crucial for the development of novel strategies to restore immunological homeostasis in inflammatory arthritis. Thus, a novel approach to creating secure and efficient arthritic therapies will come from knowing why regulatory eosinophils are induced and grown.

Indeed, it is widely documented that the predominant extrinsic reason for eosinophil growth is the helminth infection. RA is among the many autoimmune and inflammatory disorders for which several prior studies using experimental animal models have shown a clinical improvement in inflammatory activity [86,87]. These findings, together with the discovery that the glycoprotein ES-62, generated from filarial nematodes, had been demonstrated to have anti-inflammatory and anti-osteoclastogenic properties in mice models of arthritis, led to the proposal that helminths and their secreted products might constitute a feasible interventional method for the treatment of RA [88,89]. In addition, several clinical studies have been carried out to assess how helminths affect autoimmune illnesses’ immune systems, particularly inflammatory bowel disease. The host immune system is modulated by helminths and their derivatives through a variety of methods, including downregulating IFN-γ and IL-17, inducing regulatory T and B cell subsets, and changing the immunological responses from Th1 to Th2 [90]. Since the present traditional treatment of RA mostly involves non-specific immune system suppression, which frequently results in serious infections and cancers, helminth-based immunotherapy is gaining more and more attention [91]. According to this theory, one of the recent investigations revealed that collagen-induced arthritis may be effectively relieved by a little neuropeptide known as Neuromedin U, with signs of eosinophil ILC2 induction [73]. However, another method that causes proinflammatory eosinophils to differentiate into regulatory phenotypes is provided by eosinophil plasticity.

An important issue for further understanding of the mechanisms of eosinophil initiation in RA remains the study of the role of adhesion molecules such as VCAM-1 and ICAM-1 in eosinophil homing to inflamed joint areas in RA. Traditionally, VCAM-1 is known to induce eosinophil transmigration through activated endothelial cells in asthma via interaction with IL-4, which leads to increased release of granule proteins in inflamed tissues [91]. In general, VCAM-1 is also considered to act as a proinflammatory mediator in RA [91]. In this regard, it is potentially possible to discover a new role for VCAM-1 and ICAM-1 in the pathogenesis of RA associated with eosinophil homing. Understanding these processes may lead researchers to develop therapeutic agents related to these molecular mechanisms. It is known that VCAM-1 interacts with eosinophils via the CD11b receptor in asthma, which promotes their activation [92]. It is interesting to study this mechanism in relation to REs in the pathogenesis of RA.

An important issue for future research is also the research of the functions of different eosinophil populations in the pathogenesis of RA. For example, E1 eosinophils, which have shown the ability to reduce the inflammatory response in allogeneic transplantation, may be of interest [93]. Another issue for the future is research on changes in eosinophil metabolism in RA. It is known that eosinophil metabolism can change depending on the state of the cellular microenvironment, as well as during the development of various diseases [94]. This can be helped by the use of non-targeted metabolomics methods, which were used to analyze, in particular, the metabolic profile of patients with osteoarthritis [95].

## 7 Conclusions

In conclusion, fresh research indicates that eosinophils have a proresolving role in RA in addition to acting as proinflammatory effector cells. They operate in the synovium of RA patients who are in remission, and they multiply when IL-5 produced by ILC2 stimulates them. Mechanistically, regulatory eosinophils facilitate the resolution of arthritis by secreting resolvins that are reliant on 12/15-LOX and by causing synovial macrophages to change into M2 phenotypes. Since the present therapeutics for RA try to promote inflammation resolution rather than suppress proinflammatory cytokines and mediators, this understanding is essential for the development of novel strategies to restore immunological homeostasis. Among the proposed therapeutic targets, 12/15-LOX is the most promising. Future drugs aimed at enhancing the function of this enzyme have the potential to be highly effective and safe, but preclinical and clinical studies are required to provide a more robust evidence base.

## Figures and Tables

**Table 1:** Potential therapeutic strategies targeting eosinophil activation in RA

Therapeutic target	Functions in the pathogenesis of RA	Therapeutic compounds	Therapeutic mechanism of action	Features
CCR3	Activation and migration of eosinophils to joints	small molecules: CH0076989 and UCB35625 (Y113A)	CCL11 mimetics	It is not for use in case of concomitant allergies and bronchial asthma.
IL-5	Maturation and activation of eosinophils	small molecule TTK21	TTK21 activates CBP/p300 which enhances expression of IL-5	It is not for use in case of concomitant allergies and bronchial asthma.
12/15-LOX	Resolution of inflammation in joints	AKBA	Allosteric activation of 12/15-LOX	Due to its direct effect on the resolution of joint inflammation and its preferential expression in REs, 12/15-LOX is the most promising therapeutic target in terms of efficacy and safety.
		shRNA targeting KDM5C	Inhibition of KDM5C, which is a transcriptional repressor of 12/15-LOX	
Osteopontin	Synovial tissue repair	Exogenous osteopontin	Enhancement of the action of endogenous osteopontin	Selection of the optimal delivery platform to avoid the formation of Anti-Drug Antibodies (ADA).
Serpin E1	Synovial tissue repair	purinergic inhibitor P2Y12	Increasing serpin E1 expression	It is not for use in case of concomitant thrombophilia.
